# “Nothing without connection”–Participant perspectives and experiences of mentorship in capacity building in Timor-Leste

**DOI:** 10.1371/journal.pgph.0002112

**Published:** 2024-03-08

**Authors:** Jennifer Yan, Nelson Martins, Salvador Amaral, Joshua R. Francis, Barbara Kameniar, Clare Delany

**Affiliations:** 1 Menzies School of Health Research, Charles Darwin University, Dili, Timor-Leste; 2 The University of Melbourne, Melbourne, Australia; 3 The University of Tasmania, Hobart, Australia; Children’s Hospital of Eastern Ontario, University of Ottawa, CANADA

## Abstract

The literature on mentorship approaches to capacity building in global health is limited. Likewise, there are few qualitative studies that describe mentorship in capacity building in global health from the perspective of the mentors and mentees. This qualitative study examined the perspectives and experiences of participants involved in a program of health capacity building in Timor-Leste that was based on a side-by-side, in-country mentorship approach. Semi-structured interviews were conducted with 23 participants (including Timorese and expatriate mentors, and local Timorese colleagues) from across a range of professional health disciplines, followed by a series of member checking workshops. Findings were reviewed using inductive thematic analysis. Participants were included in review and refinement of themes. Four major themes were identified: the importance of trust and connection within the mentoring relationship; the side-by-side nature of the relationship (*akompaña*); mentoring in the context of external environmental challenges; and the need for the mentoring relationship to be dynamic and evolving, and aligned to a shared vision and goals. The importance of accompaniment (*akompaña*) as a key element of the mentoring relationship requires further exploration and study. Many activities in global health capacity building remain focused on provision of training, supervision, and supportive supervision of competent task performance. Viewed through a decolonising lens, there is an imperative for global health actors to align with local priorities and goals, and work alongside individuals supporting them in their vision to become independent leaders of their professions. We propose that placing mentoring relationships at the centre of human resource capacity building programs encourages deep learning, and is more likely to lead to long term, meaningful and sustainable change.

## Introduction

**Fig 1:**
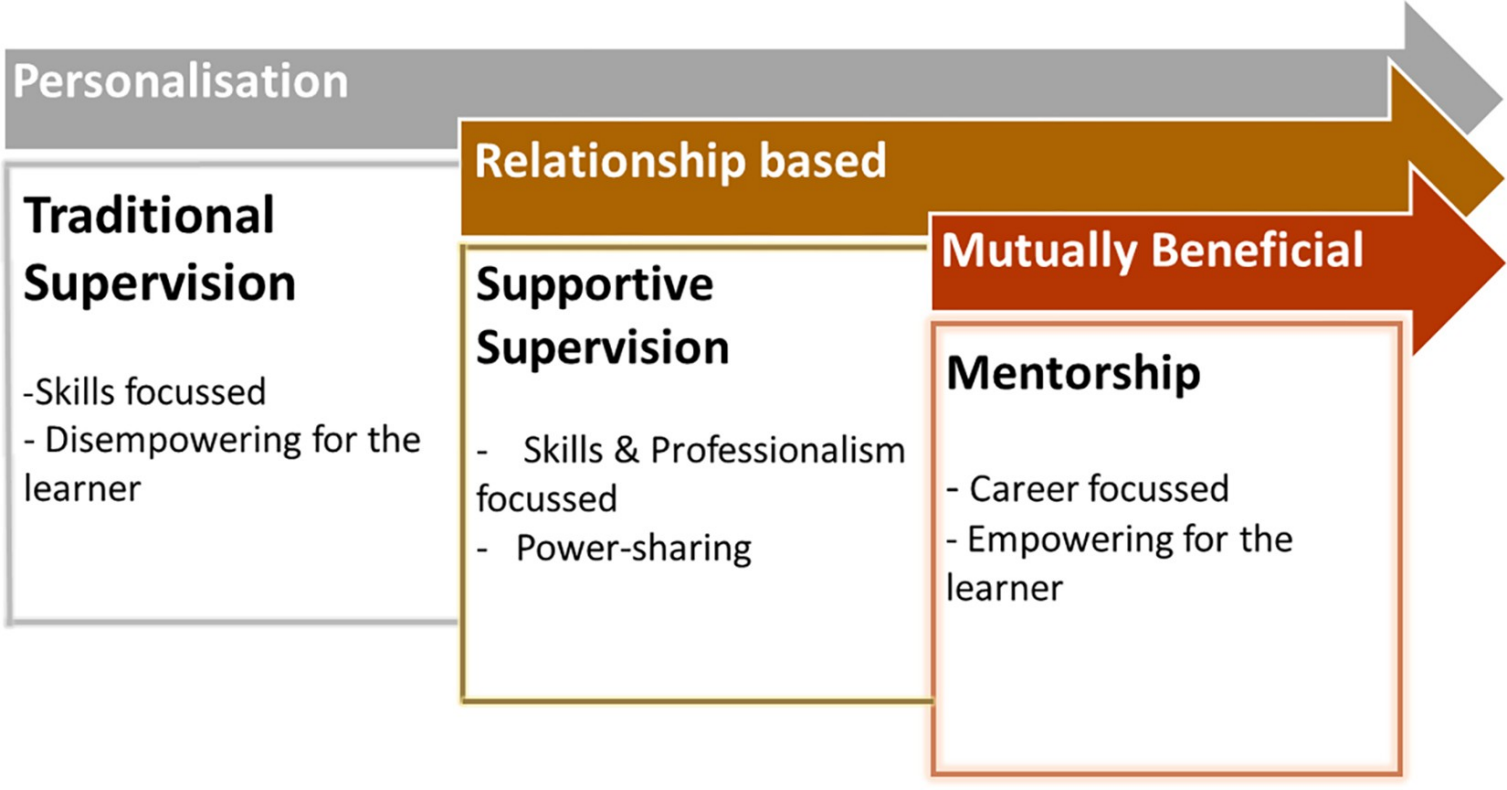
Diagrammatic representation describing the role of mentorship compared to concepts of traditional supervision and supportive supervision in global health, from Schwerdtle et al [2].

Strategies utilised for human resource capacity building in global health are many and varied, including overseas training, intensive in-country support, short course training or workshops, supervision and mentoring [1–13]. The concept of mentorship is one that is well accepted in the educational literature. The mentor is likened to an advisor or guide, with two way communication where the relationship inspires and guides learning [14]. However, in global health capacity building literature, the dominant focus is on task performance and related supervision [1, 2]; literature describing the use of mentorship is limited [1–4]. Schwerdtle, Morphet and Hall compare mentorship with familiar concepts of supervision and supportive supervision for task performance (Fig 1). They distinguish the mentoring relationship as one that shifts the focus and power balance toward the individual learner’s identified needs through respectful, collaborative relationships, two-way learning and provision of opportunities for leadership and growth [2].

Published literature evaluating mentorship approaches in global health has favoured quantitative assessment and there are few qualitative studies of participant experience [11, 15], particularly relating to mentorship in the cross-cultural setting [16–19].

This study describes participant experiences of a particular model of human resource capacity building involving intensive in-country, side-by-side mentorship with expatriate and local mentors in Timor-Leste.

## Methods

We used the Standards for Reporting Qualitative Research (SRQR) checklist when writing this article [20].

### Study setting

This study was conducted in Timor-Leste between October 2020 and November 2022. It was designed to examine the mentorship approach of a program of work that commenced in 2018, delivered by Menzies School of Health Research in partnership with the government of Timor-Leste.

Timor-Leste is located in South-East Asia, an island nation sharing a land border with Indonesia. Timor-Leste is a relatively young country, having achieved independence 20 years ago following Indonesian occupation and prior colonisation by the Portuguese [21]. Timor-Leste lost approximately one-third of its population during almost three decades of conflict, along with its infrastructure, and a generation of leaders. Timor-Leste is considered a resource-constrained setting, and is ranked 140 out of 189 countries and territories on the Human Development Index [22]. Much of the development that has occurred since independence, including the rebuilding of a national health service and health workforce, has occurred with involvement of external development partners [23].

The Menzies School of Health Research (Menzies) is an Australian medical research institute involved in delivering a program of capacity building, research and health system strengthening in Timor-Leste, working in partnership with the Government of Timor-Leste Ministry of Health, and Ministry of Agriculture and Fisheries, via projects funded primarily by the Australian and UK governments. Menzies is dedicated to improving Indigenous, global and tropical health and is registered to operate as an organisation in Timor-Leste with a local Timor-Leste office base. The Menzies program of work in Timor-Leste has focused on strengthening the country’s local health system capacity to recognise and respond to infectious diseases, which remain a major cause of morbidity and mortality [24–26]. The work is centred around a program of intensive side-by-side mentorship with mentors (expatriate and Timorese) working alongside local Timorese colleagues across a range of settings–laboratory, clinical and surveillance; and across a number of professional disciplines in human and animal health–involving doctors, nurses, public health staff, laboratory scientists and technicians, and veterinary staff. The support for human resource capacity building has occurred in conjunction with funding to support improvements in infrastructure, equipment and information management systems. Mentors are based in-country, situated in the workplace alongside their local colleagues, to exchange knowledge and skills, to model, guide, support, and help to troubleshoot application of skills into real life on-the-job practice. Most mentors work alongside a small group of local colleagues.

### Mentorship approach

The mentorship approach used in this program was informed by theoretical concepts of mentorship from educational literature and a growing understanding of the importance of decolonising global health. The role of the mentor is consistent with that described by Schwerdtle, Morphet and Hall [2]. Distinctions are often drawn between the roles of mentor and supervisor in the educational literature; however, in this program of work, mentors have a dual mentorship and supervision role, with responsibilities for workplace based training and supervision, but also a personal and professional relationship extending beyond that of a teacher or supervisor.

The approach to capacity building and expectations of the mentor role are discussed in detail as part of the staff recruitment and orientation process. Selection criteria include requirements for previous experience in a mentoring or teaching capacity, and demonstrated cross-cultural sensitivity, in addition to technical capacity. Ability to commit to at least 12 months in-country is preferred, to ensure relationships can be developed and sustained; mentor placements have ranged from 3–6 months (a minority) to over 3 years in duration.

Due to the COVID-19 global outbreak, a number of mentors were unable to continue working in-country and left Timor-Leste to continue mentoring remotely. Other mentors were able to remain in-country as their work also involved direct support of the national COVID-19 response. Two Timorese mentors returned to Timor-Leste during the COVID-19 pandemic from alternate roles overseas, specifically to support their country and the laboratory efforts at this time. We were also interested to understand the impact of this change on mentors and mentees in their experience of the mentoring relationship.

### Ethics

Ethics approval was obtained from the Human Research Ethics Committee of the Northern Territory Department of Health and Menzies School of Health Research (HREC2020-3891) and Instituto Nacional de Saúde in Timor-Leste (1684MS-INS/GDE/X/2020). A participant information sheet was provided in Tetun and English. We received written informed consent from all participants.

### Methodological orientation and theoretical framework

We took an interpretive phenomenological approach to this study. In order to centre participants’ lived experiences (learning from and with participants), we undertook a collaborative and iterative process of member checking–obtaining, checking and analysing, and drawing meaning from the data, together with participants.

### Participant recruitment and data collection

We recruited participants who had experience of the Menzies program of work either as mentors (including expatriate and Timorese mentors) or mentees (local Timorese colleagues). Potential participants were recruited by first displaying information regarding the study with flyers in the workplace, and mention of the research project at staff meetings, followed by an email distribution to potential participants and a message on a group messaging platform. Purposive sampling was used to achieve a mix of participants across professional disciplinary areas and across mentors and mentees. We had 23 participants and based on data analysis we found recurring patterns within the data sufficient to obtain data saturation.

Semi-structured interview schedules were developed by JY, reviewed together with the research team, and piloted in both English and Tetun which resulted in simplification and abbreviation of the interview schedule. We first conducted a series of individual semi-structured interviews, in which we focused on participants’ experiences and perspectives. We then held three follow-up group workshops with participants as part of a member checking process (described under data analysis). The majority of interviews were conducted in-person in a private mutually convenient location, with a few interviews conducted over online videocall. Interviews were planned to be 30–45 minutes in duration, however a number of interviews extended out to 60–90 minutes with participants keen to share their thoughts. Participants were offered the option of being interviewed in English, Tetun, or a combination of both. All interviews were audio-recorded. Interviews in English were conducted by researcher JY. Interviews in Tetun were conducted by researchers NM and SA (fluent in both Tetun and English), and later transcribed verbatim and then translated from Tetun to English, with review of the accuracy of the translation by the research team, and opportunity provided for review, clarification and edits by the participant.

The research team met together on multiple occasions to review the audio recordings and transcripts and reflect on interviews as they were conducted, discussing approaches to encourage natural conversation and ensure participants felt comfortable to share their experiences openly, including to provide negative feedback. This was predominantly achieved by revisiting the aims and intent of the research project at the beginning of each interview, sharing the motivation for conducting the research, and our desire to learn from and be guided by participant’s experiences and suggestions for how things could be done better, in designing future mentorship and capacity building work.

The data collected included audio recordings, transcripts and translations of the interviews; audiovisual recordings, translated transcripts of discussions and photographs of flip chart discussion points generated from the ‘member checking’ group participant meetings described below; and notes, observations and reflections from discussions of the research team.

### Data analysis

We used inductive thematic analysis as described by Braun and Clarke [27]. Recordings and transcripts were reviewed multiple times for data familiarisation by the principal investigator JY and initial codes generated with notes written on the text. Quotes from participants were de-identified e.g., P3 for participant 3. Coded data extracts were then collated into potential preliminary themes and subthemes. Thematic maps were used to visualise groupings of codes and subthemes under potential overarching themes and explore different linkages and interrelatedness of the data. Themes were reviewed in discussion between JY, JF, NM and SA, then further discussion and refinement of themes with CD and BK.

Inductive thematic analysis was supported and supplemented by a process of member checking to improve trustworthiness of the findings [28]. During three half day workshops with participants, draft themes were presented by the research team for feedback and discussion. Participants contributed actively to the process of thematic analysis, indicating where there was consensus and whether and how the proposed themes resonated with their story and experience, or required reframing. Transcripts from audiovisual recordings of the member checking workshops were also coded and incorporated as additional data. Participants consented to the data generated in these member checking workshops being included in the research. The member checking workshops also provided an opportunity for participants to share their experiences, learn more about mentorship, and see their input being valued, received, and heard correctly.

### Researcher characteristics and reflexivity

Authors JY (Australian, female), JF (Australian, male), NM (Timorese, male) and SA (Timorese, male) are senior members of the Menzies Timor-Leste team and are clinician-researchers with medical and nursing backgrounds. Authors CD (Australian, female) and BK (Australian, female) have not been involved in prior design or implementation of Menzies’ work in Timor-Leste in any way, and provided an external perspective, bringing additional educational and qualitative research experience to the team. JY, CD and BK have postgraduate qualifications in health professional education. Our backgrounds in clinical medicine, research, cross-cultural capacity building initiatives and medical education, and our experiences of the impacts of colonisation influenced the design of our program of health system strengthening work, and the research lens we used to explore the strength of the mentoring relationship.

All participants were known to at least one member of the research team. We recognise the potential for bias and potential for coercion in recruitment, participation, responses and interpretation of results in conducting a research study to explore participant experiences of our own program of work. We specifically sought both positive and negative feedback, and paid particular attention to recollections of negative experiences and participant suggestions for improvement. The methodology chosen was consciously collaborative and participatory, to identify the themes of greatest importance to the participant group as well as differing viewpoints, to limit the impact of views and assumptions imposed by the researchers, and to provide participants a shared forum and pathway for their suggestions to be heard and influence the program’s direction and implementation.

### Funding

There was no funding for this study.

## Results

There were 23 interviews conducted. These included 9 female and 14 male participants, and participants across human (n = 18) and animal (n = 5) health, from a range of professional disciplines including medical, nursing and pharmacy staff, laboratory scientists, surveillance and public health staff, veterinarians and veterinary technicians. There were six Australian and two Timorese mentors. A number of Timorese participants identified subsequently during the interviews with the roles of both mentor and mentee.

Participants’ answers were frank. They shared both positive and negative experiences and acknowledged many nuances to the relationship and the mentoring experience. Many participants wanted to continue the interviews over the allocated time, and to continue providing their thoughts and suggestions for improvement.

The most prominent theme from the study was the fundamental importance to participants of trust and genuine connection within the mentoring relationship itself, the foundation and centre from which deep engagement and learning was able to take place. Other key themes included the side-by-side nature of the relationship (*akompaña*); mentoring in the context of external environmental challenges; and the need for the mentoring relationship to be dynamic and evolving, aligned to a shared vision and shared goals.

Mentors and their local counterparts reflected on similar issues from different standpoints, and often their comments showed insight into the position of the other. There were no clear differences or trends across the professional disciplines in how mentorship was perceived, though experiences of mentoring relationships varied between individuals; the inclusion of a wide interdisciplinary group showed that the nature of mentorship was not specific to profession.

### Theme 1: Importance of trust, and genuine connection in the mentoring relationship—‘nothing without connection’

The word ‘connection’ was used often, and from this connection, participants described potential for deeper engagement, agreement to partner along a path of development, and deeper rather than superficial learning.

*“Mentorship stuff is about personal approach… So, having someone working closely with you, it gives you, I don’t know, a positive aura. And also… like a guarantee sort of way. Okay, I think I have someone who can help me to achieve this. I think I have someone who has this background knowledge. And I want to go there. And this is the someone. ‘Come with me.’” (P23)*

**Fig 2:**
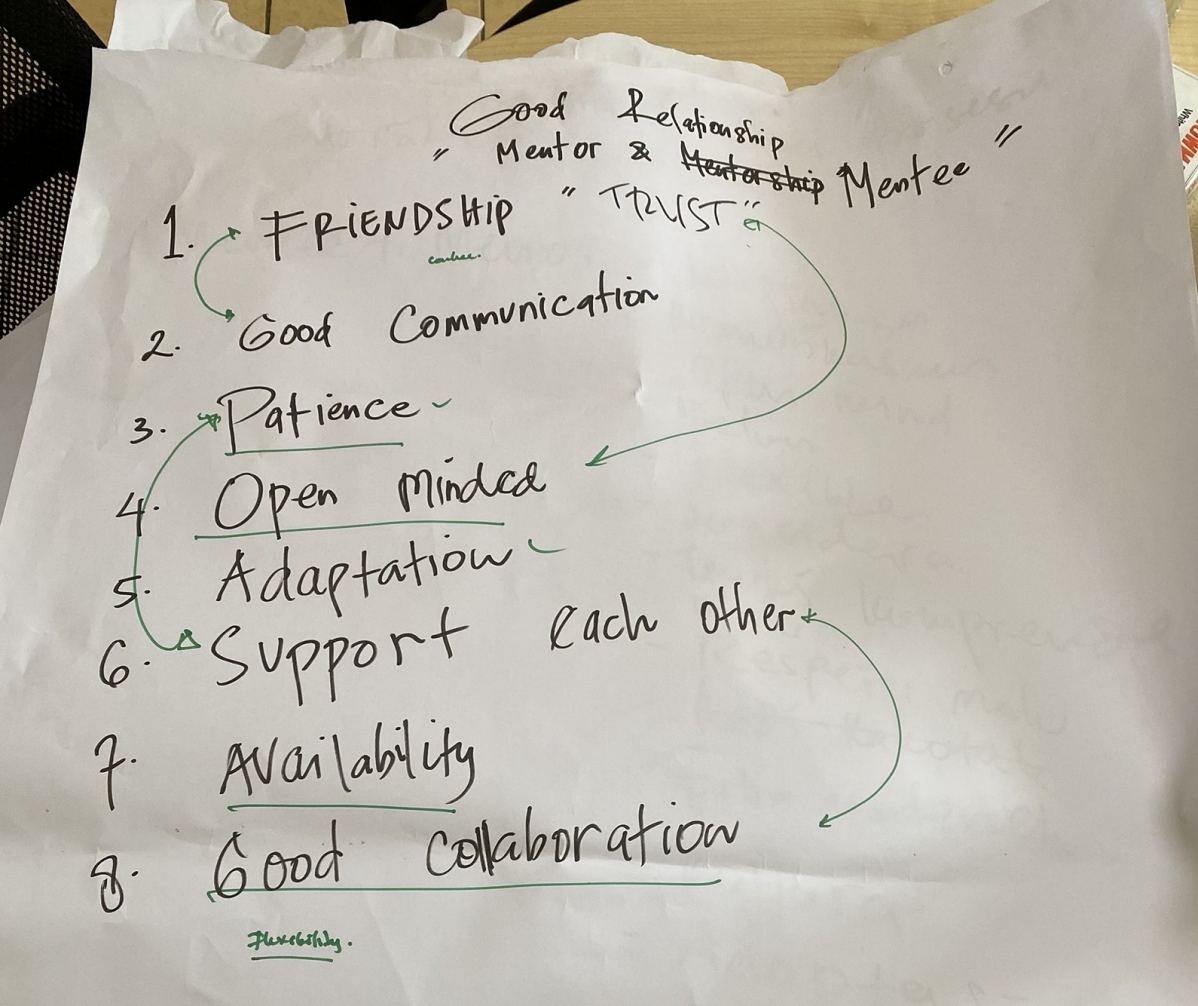
Example flip chart notes of discussion points from group participant meeting on ’what makes a good relationship between mentor and mentee’.

Participants across all areas spoke at length about the importance of the relationship, recognised by mentors as well as local counterparts, as being necessary for learning, motivation, and productive communication. Participants spoke of the necessity of mentorship being not just a professional relationship but individual connection, and friendship (Fig 2).

*“There’s no magic. Just making friends, eating food. The relationships are important… you don’t tick them off, or sign off that the relationships are going well. That’s not a, um, KPI or anything. Building relationships. But it’s important.” (P16)*

*“Side-by-side… This type of relationship, there is more confidence… instead of you’re calling doctor, he can be like a brother, when you are close… you sacrifice yourself to teach someone without return. I mean, without any payment. They respect you.” (P14)*

Timorese participants spoke about experiences they had had in other training and mentorship programs where this kind of relationship was not present, and their frustrations, the impact on their learning, motivation, sense of self-worth and achievement.

*“Especially if you come and you are the one who orders, only orders, only to teach, we feel like we are being fooled so that sometimes obstructing… work does not go well.” (P8)*

Participants emphasised that the development of this connection takes time, working together, and getting to know each other personally. Mentors reflected on the difference over time from when they first commenced in the role, and how much better they were able to support their local colleagues once they had this trust and understood the contextual challenges. They were then able to adapt their troubleshooting suggestions appropriately.

*“The first five or six months, there wasn’t a lot of output from my behalf. The first third of the project was just establishing relationships… which is an important part of mentoring. If you take time to do that properly… what’s going on now has been an accumulation of a lot of background work over years and now it’s built to a crescendo where things are firing along”. (P16)*

Timorese participants requested that mentors stay for a year, and ideally longer. In the group workshops, Timorese participants described the burden placed on them when mentors changed frequently, sometimes every six months. Adjusting to the new person and new personality, re-orientating another foreigner to the peculiarities of the local context and cross-cultural differences, introducing them to colleagues, and navigating stakeholder relationships were identified as challenges. Individual mentors would come with slightly different ways of working, and different suggestions, before coming to understand the context and history of what had come before them.

*“These conditions make us feel stressed. Because what we have learnt from first mentor is different, the second one different, the third mentor also different. Sometime it’s difficult. After one mentor came and then another mentor replaced with different system.” (P7)*

Establishing deep trust required time. Once established though, the impact of this trust on achieving a shared understanding was significant.

*“[x] is quite honest with me. So I get the real story behind things rather than the official story, or the story at face value.” (P16)*

The key finding from this study, then, was the importance of trust, connection, and commitment over the long term in the mentoring relationship, and that trust and connection take time to develop, and need to develop *in situ*. Participants reflected on the impact of the mentoring relationships on the broader program of work.

*“It’s the glue that held it together that actually made it work. If it’s not for those mentor relationships, we wouldn’t have pulled this off. These machines would have sat in isolation and not been used, and the technology wouldn’t have been incorporated… the understanding of the people that are now going to have to take this on only works because of the mentoring relationship. It would have been nice to foster even more of that to be sure that it will live on.” (P17)*

### Theme 2: The side-by-side nature of the relationship–‘*akompaña*’

When asked what mentorship meant, Timorese participants reflected that there was no direct translation for the word mentor or mentorship into Tetun, the local language. Many participants used the word ‘*akompaña’* to define their experience of mentorship, describing a sense of being accompanied, alongside, side-by-side, being motivated and supported by their mentor.

*“He is the one who always motivate us..” (P8)*

*“Like assisting with the things that we or mentor knows, the one that we do not know and to remind, motivate, give a good way the one who do not know, or to make it deeper to thing that already exist before.” (P11)*

*“More, contact, and to be side-by-side… I expect to have a mentor who works side-by-side with me.” (P14)*

This was reinforced in the group workshops. Timorese participants preferred this word ‘*akompaña’* to another word ‘*matadalan’*–also a word which means ‘to guide’, but importantly, used in a different sense of being guided ahead and following behind (rather than alongside), as one might have a guide to show you the path up a high mountain. This sentiment was also reflected by the mentors.

*"Well… you’ve got to build a bit of trust don’t you. It’s not like a teacher student relationship, like a superior inferior… is that what you call it? It’s definitely not hierarchical, I’m the boss and they’re the student. Mentoring is side-by-side. They roll their sleeves up and… like in the lab, (working) on the bench… not just throwing advice from afar." (P16)*

Both Australian and Timorese participants used similar language to describe the nature of the relationship, and it was clear that the concept of mentorship, with elements of mutual respect and two-way learning, translated across cultures. Australian mentors described needing and receiving *“a kind of cultural mentorship*.*” (P22)*.

Participants reflected on the difference between this style of long-term, side-by-side mentorship and other models of teaching and training that they had previous experience of as either teachers or learners. Whilst recognising the role that lectures and workshops could have in disseminating information to a large group of people, both mentors and local counterparts noted the limitations of the group setting on learners feeling able to ask questions, to have teaching pitched to the right level, and on translating knowledge or skills into practice. In the member checking workshops, some Timorese participants proposed that training modalities such as group lectures or workshops would be of greater value if they could be implemented alongside a follow-up schedule of regular, frequent visits for workplace-based training and support, and sharing of contact details for less formal and remote support between times. It was felt that this kind of set-up could utilise the benefits of mass information delivery while still then addressing an individual’s specific learning needs–as long as there was a consistent mentor who they could develop a trusting relationship with over that time. These descriptions articulated the key elements of support necessary to achieve the kind of accompaniment they had experienced.

**Fig 3:**
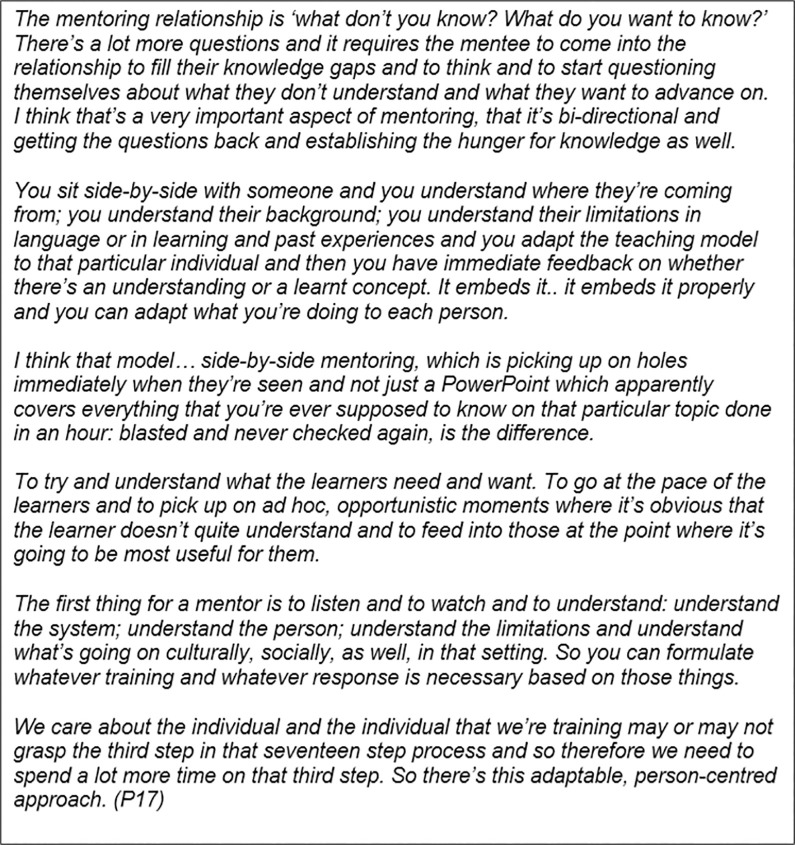
Interview excerpt from a mentor on the ‘side-by-side’ nature of the mentorship relationship, and the difference between this approach and other models of teaching, training and supervision.

Both expatriate and local Timorese participants discussed the difference it made having opportunity for Timorese to actively direct their learning journey, rather than acting as passive recipients of the ‘wisdom’ of the teacher (Fig 3). Such a disposition to teaching and learning reflects the colonial past of many new nations and is not only ineffective as a pedagogical approach, but also demeaning. Instead, mentorship as ‘*akompaña’* privileges the relationship. For participants, being ‘side-by-side’ was integral to the development and growth of the relationship itself. It also provided the context for teaching and learning through direct observation and feedback in the workplace. Participants expressed the strength of this situated learning approach as one of teaching through showing, and learning through doing, gaining apprenticeship not just of knowledge and skills, but of attitude, critical thinking and role modelling; and with the benefit of immediate application and adaptation to context. This kind of relationship required trust, and time to develop, and was considered a deeper and more personal relationship than that between teacher and learner, or with a regular work supervisor.

However, there were times when language barriers impacted effective communication and therefore the ability to effectively work alongside, and build a trusting, two-way relationship. One of the Australian mentors was fluent in Tetun; the others lamented that they had not learnt more of the local language. Participants reflected on the impact of different levels of language ability and the unintended effects this could have of limiting the potential of a mentoring relationship, excluding some people in a group from learning, or creating hierarchies of knowledge and access, or perceived favouritism within a group.

*“Colleagues were complaining us because they felt that we having no barrier to the language are the ones more close to the mentor, that is why they take some distance because they feel it is unfair to them. So they feel that the mentor just want to teach only the good one, that is why the colleagues did not want to learn because of the language barrier.” (P9)*

*“We have limitation (in language) but doesn’t mean we don’t (want to) know, we try our best to learn. As I said, language is limiting us to ask more.” (P7)*

Mentors were very aware of the issue.

*“The people that are understanding a lot of the mentoring and are part of the continuous mentoring relationship are the ones that have the most proficient English. Others unfortunately aren’t as progressed within the mentoring relationship because of the language difficulty barriers—they’re still part of the mentoring process, but the advances that you can make are limited by that. We’ve tried not to ignore them at any step, but they’re not engaged and it’s purely a language thing. It forms teams; you don’t want to have this little, secret mentoring group over here where they find out all the information, whereas the other group doesn’t find out the information.” (P17)*

Participants recognised nuance in the relationships too, and the influence of individual personalities on the success of mentorship. Attributes of a good mentor were described as being open, approachable, available, flexible, patient and humble. Timorese counterparts saw that a willingness to be open, ask, questions, and accept new knowledge were important characteristics in order to make the most from the mentoring relationship. Several Timorese participants described the contrast between an effective mentoring relationship, and other workplace relationships where knowledge may be held as power, instead of shared.

Some participants had worked alongside different mentors, and a few reported variable experiences. Those with less positive mentorship experiences described lacking strong connection, or wishing that their mentor had provided more training and support, like they saw others receiving. Contributing factors raised included different personalities and approaches to communication, a different understanding of what the mentoring relationship involved, language barriers, and lack of time.

Participants felt that the best chance of ensuring good mentoring relationships would be through careful selection of mentors with personal qualities that would be a ‘good fit’ not just for mentorship but for working cross-culturally, orientation to the role for both parties with description of intended approach and expectations of mentorship, maintaining the individualised nature of support through regular daily informal communication, regular discussions to reassess goals, and informal peer support of new mentors and mentees through regular group catch ups. This (the current approach) was supported and preferred over adoption of a more formal process using written mentorship agreements or mentorship plans, or a ‘one size fits all’ approach.

### Theme 3: Mentoring in the context of external environmental challenges

Many participants mentioned challenges outside of the mentoring relationship; structural limitations that constrained their operating environment and impacted on what mentoring and support of an individual could achieve. Within country, participants expressed having limited power to effect change within a workplace without buy-in and leadership from supervisors and leaders within government structures, including when shifts in leadership result in changing agendas and the need to establish new relationships.

*“It’s obviously clouded so much in Timor by the political nuances, the different political groups, which I still don’t quite understand and those divisions which most Timorese people utterly stand by creates conflicts behind the scenes that you don’t understand. But trying to communicate with all different stakeholders… it just can’t be done enough*.

*Having communication and creating communication channels is just so essential to success in anything. And not maintaining that can mean that you find doing things later on are difficult and remain hard.” (P17)*

Limitations of government budget also constrain available spending.

*“The government has no money. Each year when budget comes and there is available budget then we can move a bit… but after finished then we also stop, so it’s hard. We are working together with partners from [lists institutions], they sometimes come and help.” (P11)*

Other environmental factors can also influence success, especially if mentoring and training is not paired with corresponding investment in infrastructure, equipment or logistical support for people to be able to do their jobs well. Projects are limited by the interests and requirements of grant funders, and by short funding timelines, though an approach centred on mentorship could contribute to longer term sustainability beyond project end.

*“It’s hard with the way things are funded, like [project] is a ridiculous amount of money in a short time, it doesn’t allow for a lot of.. you can’t lock in mentoring. Longer term funding cycles are probably much better for this sort of [mentorship] thing, they lock in a bit of stability and longevity. I mean, part of the thing is recognizing that [if] the funding only goes for so long, that then when people develop a relationship, you still carry on supporting someone, regardless of whether there’s money or not.” (P16)*

Additionally, the global COVID-19 pandemic led to unavoidable changes in mentorship arrangements; restrictions on overseas travel of Australian citizens meant a number of Australian mentors had to leave Timor-Leste and change from working in-country to supporting remotely. This had a variable impact on their Timorese counterparts, depending on their level of skill and experience, their need for close supervision and hands on input, and the closeness of the pre-existing mentor relationship. Once the relationship was well established, online communication could still be effective, but remote mentorship was not the same as being there in person. For some, who felt ready and were competent to work independently, the distance worked well in clearly transitioning task ownership and responsibility, whilst still having regular frequent contact allowing them to feel supported and able to ask questions when needed. These participants identified advantages and disadvantages: of stepping up and not becoming dependent on the mentor’s presence and contribution to workload and decision making, but also losing the benefits of having their mentor by their side.

*“So at that time when we need something he directly responding, not even five minutes he quickly responding, so he is also doing video call, we also taking picture and sent him to have a look.. (but) when we need something that we do not yet understand, there is no one come to help for hands on to show.” (P1)*

For others, it was difficult for distance not to change the interaction with their mentor, the frequency of communication and sense of being able to ask for support.

*“The distance is making it difficult for me to have contact with my mentor. Well I think it is different. We have a doubt, we will not text it, through the email.. making subsequent questions.. so maybe big doubt, big confusion I will ask, but just asking questions… When we are close by, privately, I can ask you silly questions when we are close.. but [mentor] in Australia and me in Timor is difficult. Yeah I am not bothering them with my silly questions. Have to be big questions.” (P14)*

The experience of the participant above highlights how the side-by-side nature of the relationship was important both physically and metaphorically. Despite a relationship that had built over time, and occurred in situ, the strength of the established relationship was challenged and in this instance, disrupted by distance. Two other participants had had a mostly remote mentoring relationship (following an initial period getting to know each other in person) and described their experience from reciprocal viewpoints. They were in contact multiple times per day and video-called daily. The mentor was very available, and this supported a sense of closeness and connectedness that overcame the distance, such that it felt nearly as though the mentor was present. However, inconsistent quality and reliability of internet connection in Timor-Leste, and time zone differences, added an extra layer of challenge for participants engaging in remote mentorship.

### Theme 4: The mentoring relationship as dynamic and evolving, aligned to a shared vision and shared goals

Participants spoke of the mentoring relationship needing to be dynamic and evolving as their skills and needs changed.

*"If the person who comes to teach is based on the mapping that he will teach from A to C, but then the mentees who want to be taught their knowledge have reached D, does he have the initiative to teach from F onwards, or they will follow based on one that has been listed? So not limiting us when we want to develop, not limiting our development not feel like we do what he tells us to do. So, he gave us an opportunity.. we can developing ourselves and create our own idea.” (P1)*

Mentors recognised the need to adjust their initial preconceptions and expectations, and to change their approach and goals to fit the needs and context, not just initially, but along the whole journey.

*“Part of that is a model of also adjusting to what you see on the ground.. what was probably of significant benefit… was (to) just sit and soak up.. and then adjust a bit.. I would have envisioned doing x and more doing y.. adjusting more what we were focussing on.. other topics that were not necessarily high on my agenda going in.” (P22)*

From initial periods of listening and observation, identifying gaps and areas for improvement and developing shared goals, participants described how the relationship changed as it progressed through stages of building motivation, understanding the ‘why’ and ‘how’, modelling practice and then gradual advancement in skill toward competent and then independent practice, with changes in the level of supervision required. One mentor described the process of mentoring scientists to perform a complicated laboratory technique to a high quality assured standard.

*“You don’t expect people to learn in the first or second day you know, they take time to change the habit, take time to change the practice. Don’t just say ’I think you’ve been working on this the last five years. I think you know this’. That’s it, we miss in between. We missed the steps. Slowly showing people instead is the key. Like when we did (it). So he sat down, he did it. I sat down, I did it and compare our results. And takes time.. now they stand on their own now, now we can just stand here and they’ll do it.” (P23)*

Shared vision and shared goals were seen as necessary between mentor and local counterpart, but were also described by both sets of participants with respect to seeing their personal goals align with those of Menzies, as an organisation with which their work was involved. Both mentors and counterparts linked this with their internal motivations and described strong altruistic reasons for pursuing their line of work, for pursuing further learning and engaging with the opportunity to be involved in mentorship.

Many Timorese participants spoke of a deep motivation to serve their country and people, wanting to improve Timor-Leste, and bring dignity to the Timorese people through their work.

*“What’s motivated me is we can see the process how to help people that [are] sick. What is more important is we can help them. We can give the good results so then we can give a good treatment to the patients. Because in 2006 when the crisis (conflict) was stopped, it was only to answer the need in our country when they said there were no analysts, in laboratory and also in districts. So why they open the school and we enrolled. (P7)*

A common vision, described in participant interviews and clearly articulated in the member checking workshops, was that capacity building work should actively avoid setting up a situation of dependence. Participants (both Australian and Timorese) expressed a strong desire for Timorese to become independent in their practice, in terms of competency to perform tasks independently, and in leadership. Participants referred to the possibility of dynamic and evolving mentoring relationships, which can support a transition to independence. One participant described this eloquently as a progression via small steps from standing alongside, to standing behind, to not being needed.

*“I know it’s small, ah.. he spends time in there every day with the team, and look at them now. Like [x] stood on her own. I know that she still needs help, but they’re, they’re doing a lot of work in pandemic response. And look at (the laboratory) microbiology now. I don’t need to be there. And I know that [x] can deliver, [x] can deliver and others, others can deliver too…” (P23)*

Participants expressed their appreciation when there was an explicitly shared goal of establishing independent practice. As the mentoring relationships had evolved over time, a number of Timorese participants actively expressed a desire to move toward more distanced support, and to take the lead themselves.

*“I think their presence here… is very helpful. But on the other hand, we don’t have to depend on them forever. It is important because it doesn’t mean that we ourselves are not capable.. because one day we can. Do everything ourselves. There are things that we can do, we can decide for ourselves. For example, as now they have provided training for our infectious diseases doctor, our infectious diseases doctors are like our two friends. So far, we really need a line of people who know a little about microbiology, and the patients, and if these people are well trained, lab technicians can also make decisions in the lab even though they still need mentors from a distance. I think at times, we need [mentors].. we can do everything ourselves even though we still need them to accompany.. from afar. However, in my opinion, if in the future, we already have the best capacity… I think we are holding in a distance like the mentorship.. but I think it still needs time.” (P8)*

**Fig 4:**
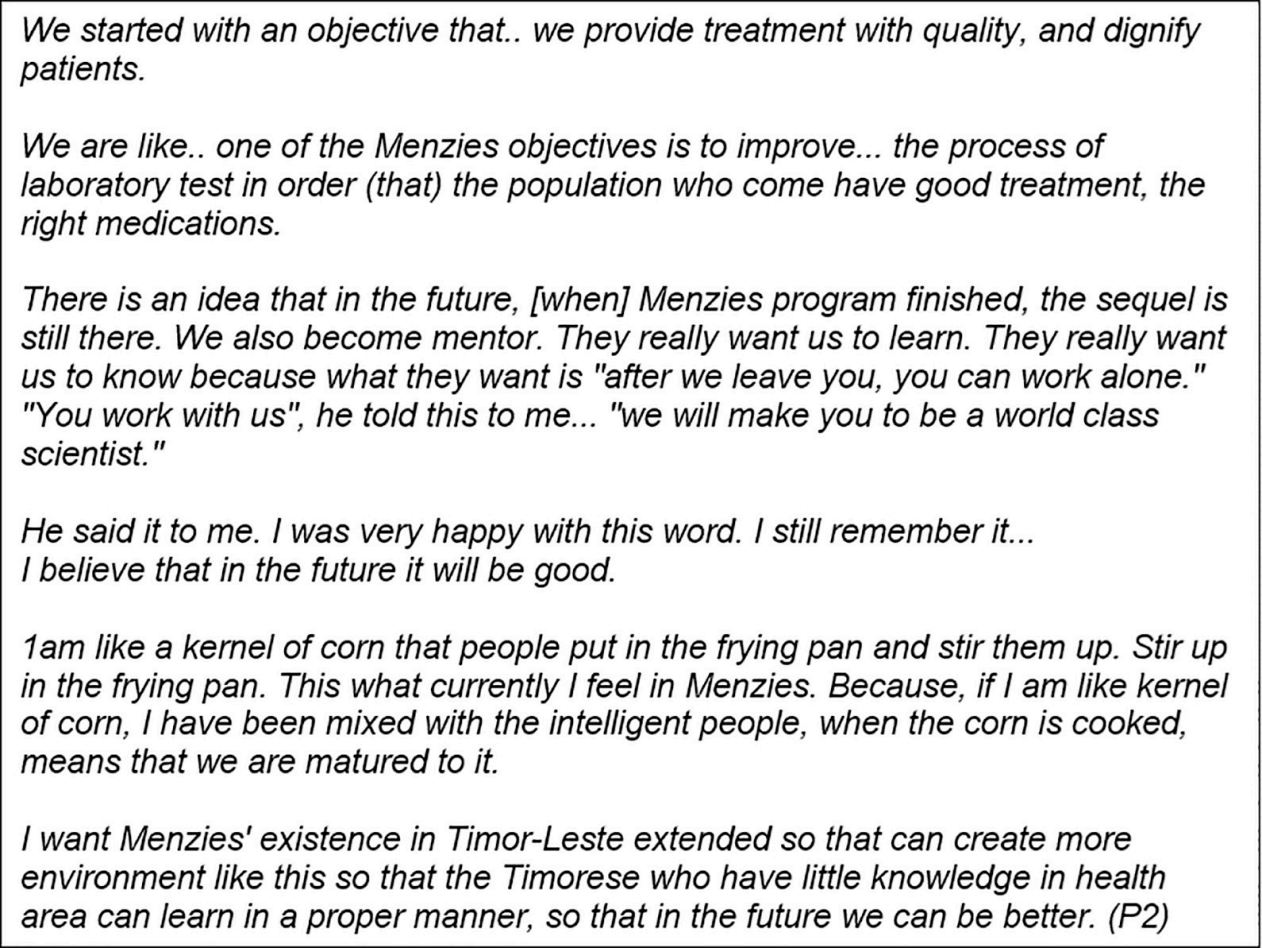
Interview excerpt from a Timorese participant on their personal motivations and a shared vision aligned with their mentor and the organisation.

Developing this shared vision represented respect, interest and investment in each individual. Those who were able to link their internal motivation with their goals from the mentoring relationship found this very motivating and powerful (Fig 4, interview excerpt).

Participants were frank about the impact of individuals and organisations where respect for Timorese leadership, capacity and vision of a pathway toward independent practice was not demonstrated. One participant described a previous experience in a different program in these terms.

*“And even some people talk, and I overheard, even in front of me, like Timor is the easy way to make money because you can just write and copy, paste, and that’s it. Nobody’s checking your report. Because people exploiting the weakness of our institution, writing technical reports, which the directors won’t even read. Now, I don’t want that to happen to my people.” (P23)*

Another shared goal was for Timorese participants to move from being mentee to becoming a mentor themselves. There was recognition of the long-term, sustained change the process enabled, and the potential ripple effect this could have upon others.

*"Every time we just rely on international consultant, consultant, consultant. We need to make sure that there is the passage of knowledge. That’s when sustainability can be achieved. So, Timor, we don’t have enough. There’s not a lot of people here in Timor who have that skill to mentor people. So we need to find a good one, so that he can replicate more good ones.” (P20)*

A number of Timorese participants already identified with both a mentee and mentor role.

*“I define mentees as a learning process to be a good mentor.*

*“What I learned from what he mentored me and what I learned from him, that I taught to other colleagues in the laboratory.” (P9)*

One Timorese participant who identified as both mentee and mentor described their personal goal in mentoring others being to mould their mentees’ attitudes and approach to knowledge sharing.

*“Knowledge is [you] need to share. There are people who are very good at something, but they are not showing, not teaching. They hold it as their knowledge. I see myself not just teaching or showing the right way to do things, but also showing the attitude and showing the character of embracing and knowledge sharing and not holding back knowledge.” (P23)*

Thus dependence and independence were spoken about in terms of task performance but also with reference to Timorese people assuming leadership, taking charge of decision making, and mentoring others. It was important that this was an explicitly shared vision between mentor, local counterpart, and the organisation, and was of particular importance given the country’s history of colonisation and struggle for independence. Some saw the continued influence of external partners, dominating decision-making by the government, as a continuation of external control.

## Discussion

The key finding from this study was the importance to participants of connection and trust in the mentoring relationship itself. Side-by-side accompaniment was crucial to development of the relationship, to obtaining a detailed understanding of specific needs and contextual factors, and to be able to provide direct and immediate support and iterative learning in a way that was respectful and founded in genuine care. Participants saw value in a personalised, tailored approach to mentorship, responsive to the changing needs and strengths of each individual over time. Participants recognised the influence of structural limitations in the broader environment in which mentorship was conducted, on what could be achieved. Cross-cultural mentoring relationships benefited from a shared vision and goal of independent practice, of particular significance given Timor-Leste’s history of colonisation and struggle for sovereign independence. The cross-cultural nature of the mentoring relationship and how this was managed was a critical thread, relevant across all themes.

We had designed our program of capacity building and health system strengthening work around mentorship as a core component of our theory of change, relying on the transfer of skills and knowledge from mentor to their local counterparts. However, the value of connection in the relationship itself was more significant to participants than we had anticipated. This was highlighted by the quote “*nothing without connection*”. It was clear that this connection, based on trust, had to develop over time, and was dependent on a period of in-country presence and the personal characteristics of the mentor.

The potential tensions between mentoring and supervisory roles are well recognised [29, 30]. Assuming responsibility for program oversight, and monitoring of quality or performance (traditional supervision) can confound the mentoring relationship, and tip the balance from a supportive and encouraging environment to one of monitoring and checking [30]. Where possible, having two different people act in the mentor and supervisor roles can relieve this conflict [31]. However, limitations of human resources can mean that one individual holds dual roles. In this situation, mentorship can be seen as ‘adding’ an additional element of support above and beyond supervision and supportive supervision [2]. In practice, the dual mentor/supervisor may hold shifting positions from encouraging and working ‘alongside’, to enforcing rules or managing performance as the ‘superior’. In this context, it is vital for mentors to maintain a strong connection and open communication to hold this duality of roles well.

The style of mentorship participants described as ‘*akompaña’*, learning side-by-side accompanied by one’s mentor, was also illustrative of a number of teaching and learning theories in practice, including situated learning [32], intentional change theory [33], and the ‘zone of proximal development’ in Vygotsky’s sociocultural theory of learning [34]. Participants described the benefits of the apprenticeship model of learning, ‘situated’ in the workplace and learning through application and adaptation of theoretical knowledge and skills to real life problems [32]. A number of Timorese participants described their personal experience of progress and development through mentorship in words that paraphrase intentional change theory which describes the conscious undertaking to develop a personal vision or goal, identify gaps between one’s current and ideal self, make a plan to move between the two, actively apply oneself to practice, and maintain relationships with people to support you to get there [33]. The close ‘*akompaña’* relationship between mentor and their local counterpart, gave mentors opportunity to observe, assess and then regularly adjust their teaching to their counterpart’s needs and level of ability. This resonates with Vygotsky’s concept of teaching to the ‘zone of proximal development’, a space just outside of a learner’s existing capacity and thus the next natural step for advancement, where they are both challenged and supported [34]. Daloz’s description of mentorship also uses the intersection between grades of challenge and support to illustrate how optimal growth can occur through mentorship [14]. Participants described a process of graded development of competency through different stages of learning, with an inverse correlation to the level of direct/indirect supervision required [35]. The end goal of mastery and independent practice was associated in our case with a desire from Timorese participants to shift roles and be able to provide the same mentorship to others.

The concept of ‘*akompaña’*, as described by participants in this study, bears similarities to the concept of accompaniment that has been associated with the work of Paul Farmer and the global health organisation he founded, *Partners in Health*. While *Partners in Health* frequently use the idea of accompaniment in the context of clinical care relationships, it has also been applied to partnerships which are similar to the side-by-side mentorship model we have adopted in Timor-Leste, including in health system strengthening and laboratory capacity building projects [36]. When Farmer talked about accompaniment, he emphasised the importance of openness and trust, a commitment to goals that are dictated by the person being accompanied, and a willingness to stay alongside for the long term, wherever the journey might lead. In a similar way, the concept of ‘*akompaña’* requires of the relationship something that extends beyond the focus areas of a particular project, or even the limited timeframe for which guidance is required. A relationship of accompaniment implies an ongoing closeness and trust, and a connection that might be expected to remain beyond the life of the project.

Though the concepts of mentorship translated across settings, cross-cultural and contextual differences highlight the need for bidirectional learning and in particular, recognition of the cultural mentorship that participants from a receiving country provide. The concept of cultural mentorship is used in Australia particularly in reference to engaging with Aboriginal and Torres Strait Islander peoples, and respecting Indigenous knowledge [37]. Cultural mentorship in the health care setting has been used to build greater understanding and to support practitioners to provide culturally safe clinical practice [37, 38]. For Timorese participants in this study, providing cultural mentorship allowed them to optimise the relationship and their own learning opportunities, by giving mentors an appreciation of context, and awareness of how and why they would need to adapt their usual practice, behaviour and communication.

Understanding participants’ experiences through a decolonising lens [39–41] gives additional perspective to participants’ reflections that for Timorese people, ‘we don’t like to be ordered about or told what to do’, and of the resistance of inaction when subjugated or disrespected, saying ‘yes, yes’ but choosing not to follow through with a plan that does not include shared aims. It was noted that there was a hierarchy sometimes implicit, sometimes overt, of deferring to a non-Timorese. Recalling the quote of one participant who bemoaned money spent on fly-in fly-out international technical advisors and copy-paste technical reports, participants were dismayed to see practices they perceived as blatantly taking advantage of their country, and of the ongoing injustice of people telling others what they should be doing in their own country.

The decolonisation of global health is “a movement that fights against ingrained systems of dominance and power in the work to improve the health of populations, whether this occurs between countries, including between previously colonising and plundered nations, and within countries” [42]. The concept of global health emerged in response to disproportional health needs between low and high income countries due to resource inequity. However, this positively framed narrative belies the reality that this disparity often exists because of a history of colonisation and exploitation of peoples, states and their resources [43], and an increasing realisation that “the operations of many organisations active in global health… perpetuate the very power imbalances they claim to rectify, through colonial and extractive attitudes, and policies and practices that concentrate resources, expertise, data and branding within high-income country (HIC) institutions” [42].

In undertaking this study, and associated work in Timor-Leste, we are mindful of the importance of decolonisation in our own setting, in our individual interactions and also in the systems and processes that institutionalise power imbalances, that we have power to influence and rectify. The findings of this study give us opportunity to reflect, and further commit to a deliberate, decolonising approach to our work in Timor-Leste. As we look to continue creating and maintaining mentoring relationships as part of our work, our aim is for mentors to support and work alongside local counterparts, seeking opportunities for development and leadership.

### Limitations

There were limitations to our study. The dual roles of researchers on this study also working within the Menzies program of work could have contributed to a sense of coercion to participate, positive bias in participant responses, and/or positively biased selection or interpretation of data in favour of reporting good participant experiences of mentorship. Participants were a self-selected group who volunteered to participate. Many had close interactions with the program and through their mentoring relationships, were known to the researchers and may have been more likely to provide positive responses. We made efforts to counter the potential for unconscious bias in thematic analysis with a participatory process, member checking, involving participants in revising and refining themes and reinforcing concepts of importance to the participants. Some interviews were conducted remotely via videocall rather than in-person. The COVID-19 pandemic interrupted progress on the research study and so some interviews were conducted at different time points through the transition between in-person and periods of remote mentorship, that could have influenced participant’s feelings about their experience.

## Conclusions

This study builds on limited published literature regarding mentorship approaches to capacity building in global health settings. Capacity building programs that focus on knowledge and skills transfer without broader considerations of teaching and learning theory and the individual needs of participants, can lead to interventions that are efficient to deliver but may result in superficial learning, poor engagement, slow transition to independent practice, and limited long-term sustainable change. Global health human resource capacity building programs should consider incorporation of the concept of accompaniment (*akompaña*) into program design, with a focus on development of trusting, long-term, side-by-side mentoring relationships, which prioritise development of a shared vision of professional goals.

## Supporting information

S1 File Alternative language abstract. Abstract in Tetun.
(DOCX)
