# Supplementary material for: “Nothing without connection”–Participant perspectives and experiences of mentorship in capacity building in Timor-Leste
Source: PLOS Glob Public Health. 2024 Mar 8;4(3):e0002112. doi: 10.1371/journal.pgph.0002112 (PMC10923460; doi:10.1371/journal.pgph.0002112)
Supplement: S1 File — Abstract in Tetun. (DOCX) [file pgph.0002112.s001.docx]

# Abstratu

Antesedente

Literatura kona-ba aprosimasaun uza mentoria ba kapasitasaun iha saúde globál ne’e limitadu. Nune’e mos, iha estudu kualitativu uitoan de'it mak deskreve mentoria iha kapasitasaun saúde globál nian husi perspetiva mentór no nia estudante (mentee) sira nian.

Metodologia

Estudu kualitativu ida ne'e ezamina ona perspetiva no esperiénsia husi partisipante sira ne'ebé envolve iha programa kapasitasaun saúde nian iha Timor-Leste ne'ebé bazeia ba aproximasaun mentoria direitamente iha rai-laran. Entrevista semi-estruturadu sira hala'o ona ho partisipante hamutuk na'in 23 (inklui mentór Timor-oan no Australianu sira, no parseiru lokál Timor-oan sira) husi disiplina saúde profisionál oin-oin, inklui mossemináriu verifikasaun ho membru lubuk ida. Rezultadu husi estudu ne’e análize uza metodu temátiku inductivu. Partisipante sira inklui iha revizaun no refinamentu ba tópiku sira.

Rezultadu

Tópiku prinsipál haat mak identifika ona: importánsia husi konfiansa no ligasaun iha relasaun mentoria nia laran rasik; natureza husi relasaun direitamente nian (akompaña); mentoria iha kontestu dezafiu ambientál externa; no nesesidade ba relasaun mentoria nian ne’ebé dinámika no evolutivu, no aliña ho vizaun no objetivu komun sira.

Diskusaun

Importánsia husi akompañamentu nu'udar elementu xave ida iha relasaun mentoria nian presiza halo esplorasaun no estudu liu tan. Atividade barak iha kapasitasaun saúde globál kontinua foka liu ba iha fornesimentu formasaun, supervizaun no apoiu supervizaun ba dezempeñu knaar kompetente. Observa husi pontu de vista deskolonializasaun nian, imperativu tebes ba autór saúde globál sira atu alinha ho prioridade no meta lokál sira, no servisu hamutuk ho individuál sira hodi apoiu sira iha sira nia vizaun atu sai lider independente ba iha sira nia profisaun. Ami propoin katak koloka relasaun mentoria iha programa kapasitasaun rekursu umanu nia klaran sei enkoraja aprendizajen ne’ebé profundu, no iha posibilidade bo’ot atu lori mudansa signifikativu no sustentavel ba tempu naruk.
